# Long-term safety of Credelio Quattro™ (lotilaner, moxidectin, praziquantel, and pyrantel chewable tablets), a novel orally administered combination endectocide for dogs

**DOI:** 10.1186/s13071-025-07201-3

**Published:** 2025-12-15

**Authors:** Kari L. Riggs, Xinshuo Wang, Scott Wiseman

**Affiliations:** 1 Elanco Animal Health, 2500 Innovation Way, Greenfield, IN 46140 USA; 2 Elanco Animal Health, Form 2, Bartley Way, Bartley Wood Business Park, Hook, RG27 9XA UK

**Keywords:** Credelio Quattro, Lotilaner, Moxidectin, Praziquantel, Pyrantel, Safety, Canine

## Abstract

**Background:**

The combination of lotilaner, moxidectin, praziquantel, and pyrantel pamoate (Credelio Quattro) is a novel systemic endectocide that provides month-long effectiveness in dogs after a single oral treatment. The safety of Credelio Quattro flavored chewable tablets was investigated when administered orally at the upper end of the recommended dosage range (20–41 mg/kg lotilaner, 0.02–0.04 mg/kg moxidectin, 5–10 mg/kg praziquantel, and 5–10 mg/kg pyrantel) and multiples thereof when administered long term.

**Methods:**

The study was randomized and blinded, with parallel groups beginning in healthy 8-week-old Beagle dogs and continuing until they reached adulthood. A total of 32 dogs were randomized among four groups (8 dogs/group) to nontreated controls or to treated groups at target doses of 1×, 3×, or 5× the maximum dose. Treatment was administered on nine occasions to dogs in a fed state every 4 weeks, with the control group receiving placebo tablets. Assessment of safety was based on regular health observations, complete physical/neurological examinations, food consumption, clinical pathology evaluations (hematology, clinical chemistry, and urinalysis), body weight, and macroscopic and microscopic examinations of collected tissues.

**Results:**

Credelio Quattro did not induce any serious treatment-related adverse effects based on health observations, physical/neurological examinations, food consumption, clinical pathology, body weight, or macroscopic and microscopic examinations. The only non-serious treatment-related effects of Credelio Quattro were a dose-dependent increase in the frequency of discolored feces, diarrhea, and vomiting (including hypersalivation associated with vomiting in two of the 5× dogs).

**Conclusions:**

This study demonstrates that Credelio Quattro exhibits a wide safety margin when administered monthly to puppies and dogs at the maximum recommended commercial dose, with only transient gastrointestinal symptoms similar to other oral antiparasitic products observed. Therefore, Credelio Quattro may be safely administered to dogs each month in accordance with the approved label.

**Graphical Abstract:**

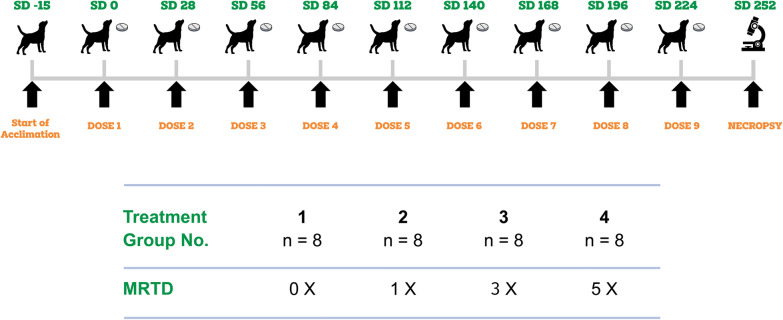

## Background

Credelio Quattro™ (Elanco Animal Health, Greenfield, IN, USA) is a new oral chewable tablet combining lotilaner, moxidectin, praziquantel, and pyrantel (pyrantel pamoate) to deliver a novel broad-spectrum endectocide for dogs. Credelio Quattro delivers considerable health benefits to dogs and peace of mind for their owners by providing protection against a broad spectrum of important internal and external parasites in a convenient monthly oral tablet. All four active ingredients (lotilaner, moxidectin, praziquantel, and pyrantel) have been widely used in veterinary medicine for years, with well-established safety profiles and dose ranges. Credelio Quattro tablets provide a minimum dosage of 20 mg/kg (range, 20–41 mg/kg) of lotilaner, 0.02 mg/kg (range, 0.02–0.04 mg/kg) of moxidectin, 5 mg/kg (range, 5–10 mg/kg) of praziquantel, and 5 mg/kg (range, 5–10 mg/kg) of pyrantel.

Lotilaner is an ectoparasiticide from the isoxazoline class, a newer group of molecules found in several similar drug products. Lotilaner has a well-established safety profile as an oral monthly chewable tablet, previously developed in mono-use products for dogs and cats (Credelio^®^/Credelio^®^ CAT, Elanco Animal Health, Greenfield, IN, USA) [1, 2] and in a combination product with milbemycin oxime for dogs (Credelio Plus^®^, Elanco Animal Health, Greenfield, IN, USA) [3]. Credelio offers proven, fast, and consistent month-long protection against fleas and ticks [4–6] for both dogs and cats and against demodectic mites (*Demodex* spp.) in dogs [7]. Moxidectin is a macrocyclic lactone (ML), the only class of molecules on the market with regulatory approval for preventing heartworm disease in dogs [8]. Owing to subtle structural differences, moxidectin exhibits unique binding to glutamate-gated chloride ion channels (GluCls) in nematodes, distinguishing it from other MLs, and thereby potentially slowing the development of drug resistance [9]. Against ML-resistant strains, moxidectin has demonstrated superior prophylactic efficacy over other MLs. Maximum efficacy against these resistant strains has been obtained by increasing both the dose and frequency of moxidectin administration [10, 11]. In 1972, Merck and Bayer developed praziquantel, a novel anthelminthic known for its broad-spectrum efficacy against parasitic trematodes and cestodes [12, 13]. In dogs, praziquantel provides comprehensive efficacy against both juvenile and adult cestodes [14]. First introduced by Pfizer in the 1960s, pyrantel is a member of the tetrahydropyrimidine family, known for its broad-spectrum anthelmintic activity against both immature and adult gastrointestinal parasites [15].

Before veterinary products can be registered or authorized, the safety of their active pharmaceutical ingredient(s) (API) must be assessed in the target species. Therefore, it was critical to determine the safety of Credelio Quattro in juvenile dogs as they are considered more sensitive owing to their rapidly changing physiology. To evaluate Credelio Quattro’s long-term safety, juvenile dogs from 8 weeks of age received doses of 1×, 3×, and 5× the upper recommended limit and continued until the dog reached adulthood when administered each month for nine consecutive 28-day treatment intervals.

## Methods

This study was a randomized, controlled, blinded study designed and conducted in accordance with the Guidance for Industry 185 Target Animal Safety for Veterinary Pharmaceutical Products VICH GL43 [16], OECD Principles of Good Laboratory Practice [17], applicable regulations of the US Food and Drug Administration (FDA) Good Laboratory Practice (GLP) Standards, 21 CFR Part 58 (5 October 1987) [18], and study protocol and testing facility Standard Operating Procedures (SOPs). Randomization was performed following the test site’s SOPs. Prior to study conduct, the test facilities’ Institutional Animal Care and Use Committee approved the study protocol. This article was prepared in compliance with the Animal Research Reporting of in Vivo Experiments (ARRIVE) guidelines checklist [19].

### Study design

Beagle dogs were randomly assigned to groups of eight (four male/four female) and orally administered Credelio Quattro or a placebo control in a postprandial state. Dosing occurred every 28 days for nine consecutive cycles at 0×, 1×, 3×, and 5× the maximum recommended therapeutic dose.

As the target species for Credelio Quattro is dogs, Beagle dogs were used as the test system. Sourced from a US Department of Agriculture (USDA)-licensed dog vendor, the study animals arrived in good health and were appropriately vaccinated by either the vendor or the test facility. Dogs considered suitable for study were housed for a 15-day acclimation period. Throughout acclimation, dogs were observed twice daily for general health, received a physical examination, and clinical pathology and fecal samples were analyzed. To ensure objectivity, the study director and all personnel involved with treatment administration and data collection were blinded to dose group assignments. This included those collecting clinical observations (veterinary physical, general, and unscheduled), body and food weights, blood samples for clinical pathology, and performing gross macroscopic examinations (i.e., necropsy). Blinding remained in effect until the conclusion of in-life, clinical pathology sample analysis processes and after assignment of Veterinary Dictionary for Drug Regulatory Activities (VeDDRA) codes to abnormal clinical observations.

### Randomization

Physical examinations were performed before dose administration commenced. Only normal, healthy dogs deemed suitable for the study were selected. This suitability was determined by evaluating their age, physical findings (e.g., weight, body condition, health observations, and physical examination results), clinical pathology results, absence of interfering behaviors, and acceptable acclimation to feeding/dosing procedures. A total of 16 healthy dogs per sex, eligible for the study, were blocked by body weight and randomly allocated to treatment groups and cages. Within each block, four dogs of the same sex were included, ensuring each treatment group appeared once. Dogs not selected for randomization or excluded from the study were transferred to the test facility’s colony.

### Treatment administration

The test article, Credelio Quattro, is a fixed combination tablet available in four sizes containing 56.25/0.056/14.25/14.25, 112.5/0.113/28.5/28.5, 225/0.225/57/57, or 450/0.450/114/114 mg of lotilaner/moxidectin/praziquantel/pyrantel (administered as pamoate salt) per active tablet. Placebo tablets were designed to bear a resemblance to active tablets and were approximately the same size.

To determine the margin of safety, multiples of the maximum recommended therapeutic dose (MRTD) are tested [16]. Specifically, the MRTD corresponds to the dose administered to the lightest-weight dog within the broadest dose range. For Credelio Quattro, the MRTD is approximately 40 mg/kg lotilaner + 0.04 mg/kg moxidectin + 10 mg/kg praziquantel + 10 mg/kg pyrantel. The MRTD was achieved as precisely as possible by utilizing single or multiple whole-tablet combinations, determined by each dog’s most recent body weight. Underdosing an individual dog by more than 10% of the MRTD was not allowed; therefore, doses surpassed the MRTD when dosing by less than 10% of the MRTD was not possible. To achieve clinical multipliers (1×, 3×, or 5×), the appropriate number and combination of tablets was administered once every 28 days for 9 consecutive months (see Table 1). Placebo tablets, equivalent in number to the 5× dose, were administered to the control group. Study day 0 marked the first day of dosing, while study week 0 encompassed the first week of dosing. Dogs were dosed in the fed state, as food has been shown to enhance lotilaner absorption [20] and does not negatively interfere with the absorption of the other three active ingredients [21, 22]. If the dogs vomited within 2 h of dosing, they were re-dosed (only one re-dose permitted per day) with either fresh tablets or expelled tablets if they were easily retrievable.

**Table 1:** Dosing scheme utilized in the target animal safety study

Dose level	Target dosage (mg/kg) for lotilaner + moxidectin + praziquantel + pyrantel received on study days 0, 28, 56, 84, 112, 140, 168, 196, and 224
0×	Placebo^*^
1×	40 + 0.04 + 10 + 100
3×	120 + 0.12 + 30 + 30
5×	200 + 0.2 + 50 + 50

^*^Control group received the same number of tablets as a dog with the same weight in the 5× group

### Food and water

Dogs were transitioned off the supplier diet (Purina^®^ ProPet Puppy Performance 30/22 (St. Louis, MO) to a certified canine diet (Lab Diet^®^ Certified Canine Diet 5L66 High Density Canine; PMI Nutrition International, Inc., Richmond, IN) during acclimation. While acclimatizing and on nondosing days, dogs had access to food for at least 12 h daily. Fasting periods were implemented before each scheduled blood collection for clinical pathology, prior to dosing and prior to necropsy. On dosing days, dogs were fed an approximate 25% portion of Royal Canin^®^ (St. Charles, MO) on the basis of the manufacturer’s recommended daily amount per individual dogs’ body weight. Following dosing, dogs received their regular daily dry food ration. Prior to the first dose, and each subsequent dose, the dogs underwent several days of acclimatation to the canned food and feeding procedure. Dogs had unrestricted access to drinking water.

### Housing and environmental conditions

During acclimation, dogs of the same sex were housed in pairs. On day 0, the first day of dosing, dogs were individually housed. Starting on day 1, dogs were housed in same-sex pairs within treatment groups. On day 28, and for the remainder of the study, dogs were housed individually. Dogs were housed in climate-controlled rooms, utilizing clean cages with stainless-steel construction and plastic-coated flooring.

### Safety evaluations

#### Health observations

Routine general health observations were performed on all dogs twice daily. Clinical health examinations were performed twice daily during acclimation, on dosing days just prior to dosing, continuously for 4 h post dosing, 10 h following dosing, and then twice daily on non-dosing days. Veterinary staff performed physical examinations on dogs at various time points including, once before randomization, once after randomization but before dosing, within the first 2 days of each dose cycle, and on the day before scheduled necropsy. The comprehensive physical examination evaluated general health and behavior, alongside a thorough assessment of the integumentary, musculoskeletal, nervous, gastrointestinal, cardiovascular, and respiratory systems. In addition, body temperature, heart rate, and respiratory rate were also measured at each assessment. Eye examinations were performed during acclimation, the day prior to dose cycles 4 and 7, and the day prior to necropsy.

### Body weights

Each dog’s body weight was measured and recorded daily during the acclimation period, twice weekly during dose cycles 1 and 2, and then weekly during the remaining dose cycles, including each day prior to dosing. The last body weight measurements, taken after fasting, occurred on the day of necropsy.

### Food consumption

Individual food weights were recorded daily during acclimation and throughout the in-life study period, with the weekly averages reported. Food consumption for each dog was calculated as g/dog/day.

### Clinical pathology

Prior to randomization, blood and urine samples were collected from all dogs for clinical pathology, including serum chemistry, hematology, coagulation, and urinalysis, along with fecal samples. Subsequent clinical pathology and fecal collections were performed 7 days post dose cycle 3 (day 63), 1 day prior to dose cycle 4 (day 83), 7 days post dose cycle 6 (day 147), 1 day prior to dose cycle 7 (day 167), 7 days post dose cycle 9 (day 231), and 1 day prior to scheduled necropsy (day 251). All blood samples for clinical pathology were collected after an overnight fast, with urine and fecal samples collected using cage pans.

The serum chemistry, hematology, coagulation, and urinalysis profiles followed standard practices, including parameters required by VICH GL 43 [16] and as described previously [3]. Fecal samples were analyzed for parasites and occult blood, with any abnormalities in color or consistency also being recorded.

### Macroscopic and histology/microscopic evaluations

Dogs were humanely euthanized via intravenous sodium pentobarbital injection followed by exsanguination on study day 252, 28 days after the final dose. A veterinary pathologist supervised the comprehensive gross and microscopic pathology assessments conducted on the tissues collected from all dogs [16].

### Statistical methods

Statistical analyses were conducted by the study statistician using SAS^®^ statistical software (Version 9.4, SAS Institute Inc., Cary, NC, USA), as described previously [3]. In brief, endpoints that were repeatedly measured and included a pre-treatment measurement (e.g., body weights and clinical pathology parameters) were analyzed using repeated measures linear mixed models (RMANCOVA). The model accounted for “treatment,” “time,” “sex,” and their associated two- and three-way interactions, and a covariate (pre-treatment value closest to dosing) as fixed effects. Block was included as a random effect in the model. An analysis of variance (ANOVA) model was applied to organ weights and organ weight ratios, which were only measured once post treatment. The fixed effects were defined as treatment, sex, and the treatment-by-sex interaction; block was a random effect. Individual comparisons of treated groups with the control depended on the significance of the treatment interaction and main effect terms. When the treatment-by-sex interaction was significant, comparisons were made within each sex. Similarly, when the treatment-by-time interaction was significant, comparisons were conducted within each time point. In cases where neither interaction was significant but the treatment main effect was, comparisons focused on averages across time and sex. The two-way interactions, main effect, and pairwise comparisons between test article groups and the control were evaluated at a 0.10 significance level. The sole exception was the treatment-sex-time interaction, which was assessed at a 0.05 significance level.

## Results and discussion

### Dose administered

At first dose administration, body weight ranged between 2.0 kg and 2.9 kg, and dogs were 55–58 days (~8 weeks) old. Dosing occurred while the dogs were in a fed condition. The target dose was 1, 3, or 5 times the nominal MRTD of 40 mg/kg lotilaner, 0.04 mg/kg moxidectin, 10 mg/kg praziquantel, and 10 mg/kg pyrantel. Fixed tablet strengths were administered, on the basis of the most recent body weights; doses were allowed to be above the target dose but were not permitted to be less than 10% below the target dose (i.e. a 1× dog could receive > 40 + 0.04 + 10 + 10 mg/kg but not < 36 + 0.036 + 9 + 9 mg/kg of lotilaner + moxidectin + praziquantel + pyrantel, respectively). Mean monthly doses are presented in Table 2 and the mean ($\pm$ SD) dose received across all dose cycles are presented in Table 3 (no adjustment was made in the calculation for doses re-administered owing to vomiting; only the original dose received is used). The average dose received across the entire study was slightly higher than the target dose for each treatment group.

**Table 2:** Mean monthly doses (mg/kg) received

API	Dose level	Dose cycle 1	Dose cycle 2	Dose cycle 3	Dose cycle 4	Dose cycle 5	Dose cycle 6	Dose cycle 7	Dose cycle 8	Dose cycle 9
Lotilaner	1×	47.9	44.5	42.3	42.1	41.1	40.6	39.6	39.8	40.1
	3×	131.5	124.5	121.9	119.3	119.0	118.8	119.0	119.6	120.3
	5×	204.3	200.4	199.5	201.3	201.4	199.7	203.4	200.3	200.3
Moxidectin	1×	0.0479	0.0445	0.0423	0.0421	0.0411	0.0406	0.0396	0.0398	0.0401
	3×	0.1315	0.1245	0.1219	0.1193	0.1190	0.1188	0.1190	0.1196	0.1203
	5×	0.2043	0.2004	0.1995	0.2013	0.2014	0.1997	0.2034	0.2003	0.2003
Praziquantel	1×	12.1	11.3	10.7	10.7	10.4	10.3	10.0	10.1	10.1
	3×	33.3	31.5	30.9	30.2	30.2	30.1	30.2	30.3	30.5
	5×	51.7	50.8	50.6	51.0	51.0	50.6	51.5	50.8	50.7
Pyrantel	1×	12.1	11.3	10.7	10.7	10.4	10.3	10.0	10.1	10.1
	3×	33.3	31.5	30.9	30.2	30.2	30.1	30.2	30.3	30.5
	5×	51.7	50.8	50.6	51.0	51.0	50.6	51.5	50.8	50.7

Dose calculations only include the initial dose offering (i.e., re-administration due to vomiting not included)

**Table 3:** Mean and standard deviation of doses (mg/kg) received across all dose cycles

API	Mean ± standard deviation dose received (mg/kg)		
	1×	3×	5×
Lotilaner	42.0 $\pm 4.2$	121.6 $\pm 5.5$	201.2 $\pm 4.2$
Moxidectin	0.042 $\pm 0.004$	0.122 $\pm 0.006$	0.201 $\pm 0.004$
Praziquantel	10.6 $\pm 1.1$	30.8 $\pm 1.4$	51.0 $\pm 1.1$
Pyrantel	10.6 $\pm 1.1$	30.8 $\pm 1.4$	51.0 $\pm 1.1$

Dose calculations only include the initial dose offering (i.e., re-administration due to vomiting not included)

No dogs that experienced vomiting within the first 2 h of dose administration had tablets that were easily recoverable and re-administered. All dogs experiencing emesis within this 2-h time frame were administered with another full dose of fresh tablets. This provided ample opportunity for the drug to be absorbed and the potential for dogs to receive a dose higher than the intended 1×, 3× or 5× MRTD. Therefore, a robust evaluation of safety could be conducted in this study, and vomiting shortly after dose administration did not impact the safety assessment.

### Health observations

On the day of dosing, vomiting shortly after test article administration in the 3× and 5× groups was most likely treatment related with vomiting occurring more frequently in the 5× dogs as compared with the 3× dogs. Hypersalivation associated with vomiting occurred in two of the 5× dogs (one dog on two different dosing days). Vomiting occurred infrequently in the 1× dogs. Diarrhea and discolored feces were occasionally observed in all treatment groups after treatment but did increase in a dose-dependent manner. In all cases, these events (vomiting, diarrhea, and discolored feces) were not considered serious or detrimental to the animal’s health, as they were transient and resolved without treatment.

No other test article-related clinical observations, veterinary physical exam findings, or ophthalmic findings were reported during the study. All other observations in the treated groups were noted with similar incidence in the control group, were limited to a few dogs or for a few days, were not noted in a dose-related manner, and/or were common findings for laboratory dogs. These other clinical observations included: skin papules, thin and/or loss of fur, skin abrasion, skin scab, thin or lean body condition score, overweight, conjunctivitis, discharge color, gingivitis, reduced appetite, salivation, teeth tartar, persistent deciduous tooth, dilated/swollen vulva, and warm to touch.

### Food consumption and body weights

While food consumption was not statistically analyzed, values appeared generally similar between control and test article groups. Body weight, which was statistically analyzed, showed no statistically significant differences between the control and treated groups.

### Clinical pathology

Hematology, coagulation, serum chemistry, and urinalysis parameters remained unaffected by the treatment. Increased bile acids were observed on two occasions (one occasion for each dog) in two 3× dogs. For one of the dogs, the bile acid value was within the normal range at the closest evaluation prior to and immediately following the abnormally high result; thus, the high value was likely a spurious finding. For the second dog, the increase in bile acids was within the normal range at the closest evaluation point prior to the abnormal result but the abnormal result occurred at the last time point evaluated, so it is unknown if the value would have returned to normal as it did in the other dog. Importantly, increased bile acids were not observed at 5× in any dog at any time point.

For all clinical pathology results, despite identifying a few statistically significant differences, these findings were not considered to have clinical importance, as there was no dose response; they lacked a correlation between sexes; showed inconsistency of change (direction and/or magnitude) in treatment groups; and/or values were within the test facility’s historical control or reference ranges. Furthermore, no clinically relevant fecal findings were attributed to treatment.

### Macroscopic and microscopic examinations and organ weights

All dogs survived until the scheduled necropsy on day 252. No treatment-related macroscopic, organ weight, or microscopic observations were found in the examined tissues. Upon macroscopic examination, all tissues examined were within normal limits. For organ weights, despite some statistically significant differences between treated groups and controls, these were not deemed toxicologically meaningful and were attributed to normal biological variation. This determination was based on a lack of dose–response relationship, similarity between organ weights when adjusted for body or brain weights, no microscopic findings to explain the weight changes, opposing effects in males and females and/or the weights were within the historical control range at the test facility.

Observed microscopic or histologic changes were deemed incidental or linked to experimental procedures (not treatment). Crucially, the treatment did not affect the prevalence, severity, or histologic character of these incidental alterations. Reproductive tissue microscopic findings were attributed to the natural maturation and growth of dogs during the study, rather than to treatment. Sporadic microscopic findings in treated dogs were not attributed to treatment administration owing to the presence of the same finding in the control animals, lack of a dose–response, lack of consistent finding between males and females, and/or presence of the finding in CRL’s historical control database. For example, minimal mononuclear cell infiltration of the liver was noted microscopically in five control dogs, two 1× dogs, three 3× dogs, and five 5× dogs. As the number of dogs affected was the same in control and 5× dogs, this was not considered treatment related within the study. As another example, one control dog, one 1× dog, two 3× dogs, and none of the 5× dogs also had minimal extramedullary hematopoiesis. This finding did not occur in a dose-dependent manner, as it did not occur in the 5× dogs and, thus, was not considered treatment related within the study.

### Treatment considerations

Globally, dogs face a variety of risks from endo- and ectoparasites. Fleas, ticks, filarial parasites, cestodes, and gastrointestinal nematodes may present a year-round threat, depending on the dog’s lifestyle, geographic location, and local parasite pressure. Credelio Quattro, as a broad-spectrum endectocide, provides pet owners and veterinarians with a safe and effective treatment option that supports the recommendations from various scientific expert groups around the globe. The Companion Animal Parasite Council, the European Scientific Counsel Companion Animal Parasites, and the Tropical Council for Companion Animal Parasites recommend regular treatment and control of flea, tick, and gastrointestinal parasites and heartworm prophylaxis that could include monthly year-round administration on the basis of the dogs’ risk [23–25]. In total, the findings in this safety study confirm that Credelio Quattro can be safely administered monthly to dogs, as per its approved label and in support of the recommendations of parasite expert groups.

## Conclusions

This study demonstrates that Credelio Quattro exhibits a wide safety margin when administered monthly to puppies and dogs at the maximum recommended commercial dose. A long-term safety investigation involving careful clinical examinations, clinical pathology assessments, and macroscopic/microscopic examinations concluded that the combination oral chewable tablet of lotilaner, moxidectin, praziquantel, and pyrantel (Credelio Quattro) is well tolerated. Administration for nine consecutive months, starting in juvenile dogs, at doses up to 5× the maximum recommended dose resulted in no serious treatment-related effects.

## Data Availability

Data supporting the main conclusions of this study are included in the manuscript.

## Abbreviations

ANOVA: Analysis of variance
API: Active pharmaceutical ingredient
ARRIVE: Animal Research Reporting of in Vivo Experiments
CFR: Code of Federal Regulations
CYP: Cytochrome P450
FDA: US Food and Drug Administration
GLP: Good Laboratory Practice
LLC: Limited Liability Corporation
ML: Macrocytic lactone
MRTD: Maximum recommended therapeutic dose
OECD: Organization for Economic Cooperation and Development
RMANCOVA: Repeated measures analysis of covariance
SOPs: Standard operating procedures
US: United States
VeDDRA: Veterinary Dictionary for Drug Regulatory Activities
VICH: Veterinary International Conference on Harmonization
